# The Impact of Thyroidectomy and Lobectomy on Patients’ Health-Related Quality of Life, Eastern Region, Saudi Arabia

**DOI:** 10.3390/clinpract14040101

**Published:** 2024-06-29

**Authors:** Zainab Ali AlOsaif, Hassan Mohammed Al Bisher, Hend Abdelmonem Elshnawie, Mohammed Taha Al-Hariri

**Affiliations:** 1 Dammam Medical Complex, Ministry of Health, Dammam 32617, Saudi Arabia; zalosaif@moh.gov.sa; 2 Department of Surgery, College of Medicine, Imam Abdulrahman Bin Faisal University, Dammam 34211, Saudi Arabia; hmalbisher@iau.edu.sa; 3 Fundamental of Nursing Department, Nursing College, Imam Abdulrahman Bin Faisal University, Dammam 34211, Saudi Arabia; haalshnawie@iau.edu.sa; 4 Department of Physiology, College of Medicine, Imam Abdulrahman Bin Faisal University, Dammam 34224, Saudi Arabia

**Keywords:** thyroidectomy, health-related quality of life, benign, malignant, Saudi Arabia

## Abstract

Introduction: The thyroid gland is a crucial endocrine organ that can be susceptible to various pathological conditions, often necessitating total thyroidectomy or lobectomy. It is a common surgical procedure in Saudi Arabia. Thus, it is essential to recognize the impact of thyroid surgery on patients’ health-related quality of life (HRQoL). Aim: The aim of this study was to evaluate HRQoL among patients with benign and malignant thyroid diseases who underwent thyroidectomy in the eastern region of Saudi Arabia. Methods: This cross-sectional study was conducted at King Fahad Hospital of University in Khobar, Kingdom of Saudi Arabia from January 2018 to May 2018. The data collection method used was divided into two parts. Part I included the patients’ socio-demographic characteristics and the clinical characteristics of their thyroid surgery. Part II was a HRQoL questionnaire (SF-36, version 1.0). Results: The sample included 100 thyroidectomy patients ≥18 years. The study findings revealed that the overall scores for post-thyroidectomy patients showed a significant improvement in HRQoL, and HRQoL was not significantly associated with benign or malignant thyroid diseases. Conclusion: Especially when the surgery is performed by a high-volume endocrine surgeon, thyroidectomy may lead to significant improvements in HRQoL among patients, including the elderly and younger adults, with benign and malignant thyroid disorders. There was no difference in HRQoL between patients who underwent total thyroidectomy or thyroid lobectomy. Also, HRQol among thyroidectomy patients is associated with their educational and occupational statuses.

## 1. Introduction

Globally, thyroidectomy is a widely performed surgical procedure, involving the removal of all or part of the thyroid gland. It is employed as the primary treatment modality for both benign and malignant thyroid diseases, including symptomatic goiter, hyperthyroidism, and thyroid cancer [1]. Nevertheless, despite the high rate of long-term life expectancy after thyroidectomy, health-related quality of life after surgery has still not been satisfactorily clarified [2].

Health-related quality of life (HRQoL) is “a multi-dimensional concept that includes domains related to physical, mental, emotional, and social functioning”. It is influenced by disease status, its management, and its associated complications [3].

Several studies have reported that thyroid surgeries have increased over the past three decades, and they are more common among females than males. In the United States, between 118,000 and 166,000 patients undergo thyroidectomy each year for both malignant and benign diseases [4].

Thyroidectomy and lobectomy have been extensively studied for their impact on quality of life. Research has consistently demonstrated that patients undergoing total thyroidectomy generally report poorer self-assessment scores compared to those undergoing lobectomy, regardless of tumor histological subtype or stage. Furthermore, hypothyroidism requiring levothyroxine therapy has been linked to a significant negative impact on quality of life, particularly in terms of fatigue, emotional well-being, and cosmetic concerns [5].

In Saudi Arabia, thyroid gland disorders are prevalent among adults. In addition, they are the second most prevalent endocrine disease, behind diabetes mellitus, and occur in 3% to 5% of the population.

Cancer of thyroid gland is a significant health concern in Saudi Arabia, particularly among women, where it ranks second in terms of incidence. For men, it ranks tenth. According to a report, the annual incidence of thyroid cancer among Saudi nationals is approximately 1323 cases. The age-standardized incidence rate (ASR) for thyroid cancer in Saudi Arabia is 10.6 per 100,000 for women and 2.9 per 100,000 for men, indicating a higher incidence among women compared to men [6]. This finding is in line with numerous studies that have documented a high prevalence of thyroid cancer in Saudi Arabia [7,8].

As a result, thyroidectomy is a common surgical procedure in Saudi Arabia [8]. Moreover, in one study conducted in Saudi Arabia, total thyroidectomy was performed in approximately 93% of 600 patients with differentiated thyroid cancer [9].

The existing literature on the impact of benign and malignant thyroidectomy on patients’ HRQoL in Saudi Arabia highlights the importance of considering various factors that influence HRQoL outcomes [10]. These factors include the type of surgery, pathology, gland volume, and other complications such as RLN injury. These studies also emphasize the need for interventions and support to address the challenges faced by thyroidectomy patients and to optimize their HRQoL.

While the morbidity and mortality rates associated with thyroidectomy are relatively low, there are persistent long-term postoperative complications that continue to pose substantial health and social challenges for patients that affect their quality of life. Studies have shown that thyroidectomy can impact bone metabolism, thyroid hormone levels, and body mass index, especially in hypothyroid patients, highlighting the intricate relationship between the thyroid gland, hormones, and overall physiological functions [11].

On the other hand, a study comparing HRQoL in patients who underwent thyroid lobectomy versus total thyroidectomy revealed that the two groups had comparable HRQoL scores, suggesting that the extent of surgery may not significantly impact HRQoL. [12]. These findings highlight the importance of considering the type of surgery and its potential effects on thyroid function and overall well-being when evaluating the HRQoL of thyroidectomy patients.

There are variations in the specific challenges faced by patients post thyroidectomy in Saudi Arabia, and it is clear that HRQoL is a significant concern. Prior Saudi studies that contain designs to assess HRQoL in thyroidectomy patients reported that participants experienced difficulties related to their senses, body satisfaction, sexual satisfaction, eating, speaking, and social interactions [10,13]. Meanwhile, another documented that the overall HRQoL was found to be satisfactory [14].

For that reason, an accurate understanding of the treatment modality’s influence on patients’ HRQoL aspects is essential [8]. Nevertheless, HRQoL after thyroid surgery among patients with thyroid disorders remains to be clarified because there are studies that showed improvement in HRQoL after thyroidectomy. By contrast, other studies showed significant impairment in HRQoL among patients with some thyroid diseases. Furthermore, there is a lack of studies that explore the impact of thyroidectomy on HRQoL in Saudi Arabia, especially in the eastern province. Thus, this study aimed to evaluate the level of HRQoL among patients with benign and malignant thyroid diseases who underwent thyroidectomy.

## 2. Materials and Methods

### 2.1. Study Design and Setting

A cross-sectional cohort study was conducted from January 2018 to May 2018 at the endocrine surgery clinic of King Fahad Hospital of University in the eastern province of Saudi Arabia. The allocated duration of our cohort study was deliberately chosen to capture both short-term and long-term postoperative outcomes. This duration allows us to assess immediate postoperative complications and recovery, as well as the long-term effects on quality of life, functional ability, and overall well-being, as reported in prior reports [15,16].

Some patients underwent total thyroidectomy, while others underwent thyroidectomy with central neck dissection. Additionally, some patients underwent total thyroidectomy with both central neck dissection and lateral neck dissection. Neck dissection is recommended for patients with nodal involvement detected pre- or intraoperatively. It is also recommended for patients with clinically node-negative disease who have a high risk of nodal metastases [17].

All patients who met the inclusion criteria were enrolled in the study.

### 2.2. Inclusion Criteria

- Patients older than 18 years old
- Patients who were able to complete the HRQoL questionnaire
- Patients who underwent thyroidectomy and lobectomy due to:
- Differentiated thyroid cancer with no metastasis
- Obstructive symptoms/goiter causing dyspnea or dysphagia
- Suspicious thyroid nodules along with hyperthyroidism
- Hyperthyroidism along with huge goiter
- Hyperthyroidism contraindicated for radioisotope scanning and ablation

### 2.3. Exclusion Criteria

- Patients with thyroidectomy who underwent a recent operation or those who underwent an operation within the past month.
- Patients not operated on by an endocrine surgeon.
- Patients with psychiatric disorders and a history of brain injuries who are unable to participate.

### 2.4. Participants of the Study

A total of 100 thyroidectomy patients who fulfilled the inclusion criteria were selected randomly from the endocrine surgery clinic at King Fahad Hospital of University.

Step 1: Sample size SS = Z2 * (*p*) * (1 − *p*) C2

Where Z = 1.96, *p* = 0.5

C = Confidence interval = 0.05 SS = (1.96)2 * 0.5 * 0.5/(0.05)2

SS = 384.16.

Step 2: Sample size to the corresponding population size

Final sample size = SS/[1 + (SS − 1)/Population], population size = 120 sample size = 384.16/[1 + (384.16 − 1)/120

Therefore, the final sample size in relation to the corresponding population = 91.68. Thus, it is approximately *n* = 100.

### 2.5. Data Collection Tool

The method used for data collection in this study was divided into two parts.

*Part I.*

*Section I.*

This section addressed the patients’ socio-demographics, which included information on gender, age, nationality, marital status, education, and employment status.

*Section II*.

This section addressed the patients’ clinical characteristics of thyroid surgery, which included an indication for thyroidectomy, the type of thyroidectomy, and the follow-up duration after the surgery.

*Part II.*

HRQoL questionnaire, which is the Short-Form 36-Item Health Survey (SF-36, version 1.0). The SF-36 survey is an internationally accepted general measure of HRQoL with excellent validity and reliability [18]. The Arabic and English versions were used. It consists of 36 closed-ended structured questions to assess HRQoL, grouped into eight domains: general health, physical functioning, social functioning, physical, vitality, bodily pain, emotional, and mental health). Each domain involves several numbers of questions [19].

The first domain, physical functioning, contained 10 questions. The second domain, role limitations due to physical health, contained 4 questions. The third domain, role limitations due to emotional problems, contained 3 questions. The fourth domain, energy/fatigue, contained 4 questions. The fifth domain, emotional well-being, contained 5 questions. The sixth domain, social functioning, contained 2 questions. The seventh domain, pain, contained 2 questions. The eighth domain, general health, contained 5 questions.

Additionally, a self-reported health transitional item is included, which is completed by the client but not factored into the scoring. Each domain’s score is then transformed into a scale ranging from 0 (worst possible health status) to 100 (best possible health status).

### 2.6. Permissions and Ethical Approval

Ethical approval was obtained from Imam Abdulrahman Bin Faisal University Ethical Committee in March 2018 (IRB no. PGS-2018-04-062). After that, official permission from the director of King Fahad Hospital of the University was obtained to conduct the study. Moreover, informed consent was obtained from each patient through both verbal and written means.

### 2.7. Statistical Analysis

The statistical analysis of the data and the data presentation was conducted using the IBM SPSS Statistics program (version 23.0). However, after the questionnaire data were collected, they were coded and inserted into the SPSS program. Continuous data were presented as means with standard deviations, while categorical data were presented as frequencies with percentages.

The total median scores for each category were presented using a bar diagram. Also, the association between the overall total mean scores and socio-demographic variables with clinical characteristics of thyroid surgery were tested using an independent *t*-test and ANOVA test. In addition, significant associations between the socio-demographic variables, clinical characteristics of thyroid surgery and total scores of each category, were tested using the Mann–Whitney U test and Kruskal–Wallis test. The threshold for statistical significance was set at *p* < 0.05.

## 3. Results

A total of 100 thyroidectomy patients who fulfilled the inclusion criteria were selected from the endocrine surgery clinic at King Fahad Hospital of the University.

Table 1 illustrates the distribution of patients’ socio-demographic characteristics and shows that 80% of the studied patients were females out of a total of 100 thyroidectomy patients. With regard to the age groups of the studied patients, about half of the studied patients (52%) were 41–60 years old, 38% were 21–40 years old, and 8% were >60 years old. Regarding nationality, 91% (*n* = 91) of the studied patients were Saudi, and regarding marital status, 80% of the studied patients were married. With regard to job categories, the majority of studied patients (65%) were unemployed. Finally, regarding educational status, 22% of the studied patients received an elementary education, 21% received a secondary education, less than half (43%) had a bachelor’s degree, and only a few had master’s (3%) and doctorate degrees (5%).

**Figure 1:**
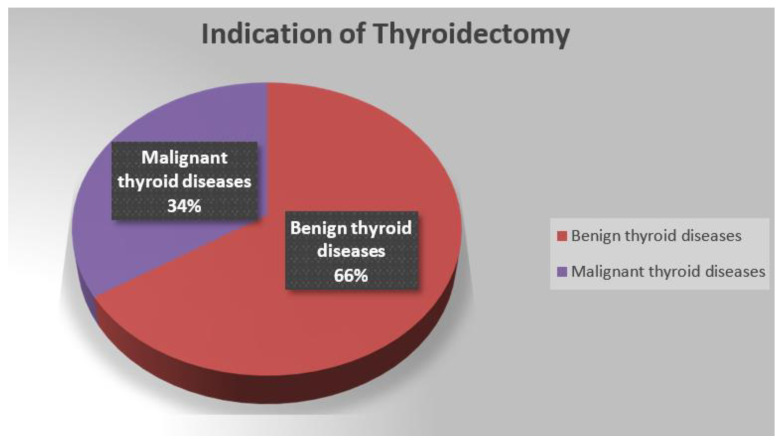
Distribution of patients with thyroidectomy according to their thyroidectomy indication.

**Figure 2:**
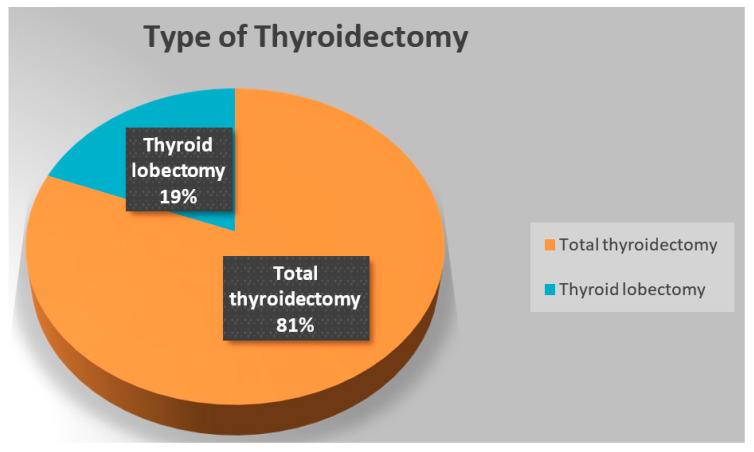
Distribution of the studied patients according to the type of thyroidectomy.

Figure 1 shows the distribution of the studied patients according to clinical characteristics of thyroid surgery. It illustrates that 66% of thyroidectomy indications were benign thyroid diseases and 34% were malignant thyroid diseases. Furthermore, 81% were total thyroidectomies (Figure 2).

Table 2 shows the overall total median percentage scores for the quality of life of the patients who underwent thyroidectomy. The quality of life domains are listed: role functioning/emotional, role functioning/physical, pain, and social functioning. Related quality of life data had a median of 100, and the remaining four items had a median of ≥80. The score of each domain ranges from 0 (worst possible health status) to 100 (best possible health status). Thus, the quality of life was enhanced after thyroid surgery.

**Part II**

The second section of the results was conducted to evaluate the correlation between the socio-demographic or clinical characteristics and the overall total mean scores.

Table 3 illustrates the association between the socio-demographic or thyroid characteristics and the overall total mean quality of life scores for the studied patients. It shows that there was a statistically significant positive correlation between the occupation (*p* = <0.002) and education (*p* = <0.001) variables only. It also indicates that an employed status and a higher education level showed a significant positive correlation with the mean quality of life scores for the studied patients.

Table 4 displays associations between the median percent general health scores of the studied patients and their socio-demographic or thyroid characteristics. The results indicate that there is no statistically significant relation between the variables.

## 4. Discussion

To the best of our knowledge, the current study is the first to use HRQoL scores for thyroidectomy patients in the eastern province of Saudi Arabia. The results of this study showed that the majority of patients in this study had benign thyroid diseases, and out of all the thyroid surgeries, total thyroidectomy, which is a common surgical intervention, predominated. Interestingly, the overall postoperative HRQoL scores obtained from patients after thyroidectomy were high in both benign and malignant disorders.

Surprisingly, in contrast to our expectations, the results did not show a significant difference in HRQoL between patients with benign and malignant thyroid disorders, as we hypothesized that thyroid cancer patients would have a lower HRQoL based on previous studies. Prior reports have shown that patients with thyroid cancer may experience challenges with HRQoL post surgery, especially in the short term, with variations based on the extent of the surgery. For instance, HRQoL tends to be higher after hemithyroidectomy compared to total thyroidectomy or total thyroidectomy with central neck dissection in patients with differentiated thyroid cancer [20].

In agreement with the present study, recent studies found no difference in HRQoL between patients with benign and malignant disorders post thyroidectomy [21,22]. Meanwhile, the lack of social support for patients who experience postoperative complications and recurrence have been previously recognized as an important driver of poor HRQoL among survivors of thyroid cancer [23,24]

Thus, we assumed this might be attributed to the quality of the health services and surgical procedures or surgical care, combined with the professional skills of the surgeon (e.g., a high-volume surgeon vs. a specialized endocrine surgeon) who carried out the surgery.

Interestingly, we found in the present study that a higher education level showed a significant positive association with the mean HRQoL scores of the studied patients. Studies have consistently shown that patients with higher educational levels tend to have better HRQoL outcomes, likely due to a greater understanding of their condition and an ability to manage their treatment effectively [25,26]. Higher education is significantly associated with an enhanced quality of life, encompassing various dimensions such as economic stability, health, and social well-being. Studies indicate that individuals with higher education levels tend to secure better-paying jobs, leading to increased financial security and access to superior living conditions [27]. Furthermore, higher education is linked to improved health outcomes, as educated individuals are more likely to engage in health-promoting behaviors and have access to better healthcare services [28].

Similarly, patients with stable employment tend to experience a better HRQoL compared to those without stable occupations. Employment is a critical determinant of HRQoL, especially for individuals with chronic illnesses or disabilities [29]. Maintaining employment can help provide financial stability, social connections, and a sense of purpose, all of which are important for overall well-being and quality of life [30]; this may be related to the reduced financial and emotional stress associated with stable work [31]. A study found that employed thyroid cancer survivors scored significantly better compared to those who were unemployed regarding various quality of life outcomes, including overall quality of life, voice problems, and neuromuscular problems [32]. These findings highlight the importance of considering the socioeconomic factors that impact HRQoL in thyroidectomy patients, particularly in the context of developing effective postoperative care programs to optimize their long-term outcomes.

Previous studies reported poor HRQoL scores among patients with thyroid disorders. Sorensen et al. reported that patients with goiters had poorer HRQoL scores compared to the general population. However, after thyroid surgery, significant improvements were observed in anxiety, fatigue, and goiter symptoms as well as overall HRQoL [33].

However, previous authors of similar studies confirmed our assumption. Zivaljevic et al. revealed that when a thyroidectomy is performed by an experienced endocrine surgeon, there is a lower risk of complications compared to when the same procedure is performed by a general surgeon [2]. Moreover, another study indicated that a surgeon volume threshold (>25 total thyroidectomies/year) is associated with improved patient outcomes [1].

Concerning age-related morbidity in thyroid surgery, the literature presents varying data. However, a previous study suggested that thyroid surgery in patients aged 70 years or older is generally safe, with age alone not being a major consideration for surgery. While some research suggests that older age does not significantly impact postoperative morbidity and mortality in thyroid surgery [34], other studies highlight an increased risk of complications in elderly patients, particularly those over 80 years old, due to factors like cardiovascular, respiratory, or urinary issues [35]. Despite the mixed data, individualized risk assessments and careful preoperative evaluations are crucial for optimizing surgical outcomes in older adults undergoing thyroid surgery [34].

Collectively, age appears to have a negligible influence on the outcome of thyroid surgery [36]. However, the findings of the present study showed that HRQoL scores did not have a significant association with age groups. This seems to be consistent with other researchers. For example, Tabriz et al. showed that surgical intervention before the age of 70 years can reduce the risk of complications and improve HRQoL regardless of patient age [37].

On the other hand, in our study, even though there was no association between the socio-demographic features or characteristics of thyroid surgery in thyroidectomy patients and their general health. There is no conclusive evidence to support a direct association between socio-demographic or clinical characteristics of thyroid surgery in thyroidectomy patients and their general health. For instance, prior studies found that socio-demographic and thyroid characteristics were not significantly related to a higher improvement in the HRQoL of thyroid surgery patients [38,39], but other studies did show a significant relationship between these variables. However, several influential factors may contribute to these relationships, such as the surgeon’s volume, hospitals, and incomes [40,41].

Previous reports showed that thyroid lobectomy has fewer negative effects on HRQoL than total thyroidectomy. In contrast to their findings, our study had more patients who underwent total thyroidectomy vs. lobectomy and showed no significant difference in HRQoL among thyroid lobectomy or total thyroidectomy. The available data do not support the premise that the surgical procedure itself (total thyroidectomy vs. lobectomy) has a predominant effect on HRQoL that can be attributed to the surgeon’s skills [42].

The general health and overall HRQoL of the patients in this study were good. A similar study analyzed the impact of thyroidectomy on general health post thyroidectomy in patients with thyroid carcinoma [14].

The significant improvement in HRQoL among the study patients with benign and malignant thyroid disorders can lead to increased patient satisfaction with their treatment outcomes, which is crucial for patient-centered care. This satisfaction can foster trust and cooperation between patients and healthcare providers, ultimately improving treatment adherence and overall outcomes [43]. Furthermore, improved HRQoL can help clinicians identify and address postoperative complications more effectively, informing strategies for managing complications and improving patient outcomes. By prioritizing patient-centered care, personalizing treatment strategies, and addressing HRQoL issues, healthcare providers can improve patient outcomes and overall well-being [44].

Importantly, enhanced HRQoL can significantly contribute to advancements in diagnostic techniques. More importantly, improved HRQoL can lead to more accurate patient-reported outcomes, which are crucial for effectively identifying and addressing symptoms and complications. This can inform the development of more targeted and effective diagnostic strategies. Furthermore, improved HRQoL can help clinicians identify individual patient needs and preferences, enabling personalized treatment approaches that are more likely to address specific symptoms and improve overall outcomes. Additionally, improved HRQoL can foster more active patient participation in the diagnostic process, resulting in more comprehensive and accurate diagnostic assessments. Moreover, improved HRQoL can contribute to the development of standardized outcome measurement tools, ensuring that diagnostic assessments are consistent and comparable across studies and clinical settings [45].

## 5. Conclusions

Thyroidectomy may lead to significant improvements in HRQoL among patients, including the elderly and younger adults, with benign and malignant thyroid disorders. There was no significant difference in HRQoL between patients who underwent thyroid lobectomy and those who underwent total thyroidectomy, especially when the surgery was performed by a high-volume endocrine surgeon. Also, the study showed that HRQoL among thyroidectomy patients is associated with patients’ education and their occupational status.

The limitations of this study include the cross-sectional design, which does not allow for the establishment of a clear temporal association between the exposure and the outcome. Additionally, the study’s recruitment process may have introduced biases, as the sample was not randomly selected. The data collection methods used in this study may also have introduced biases, such as those related to self-reported data or measurement errors. Finally, the study’s findings may not be generalizable to other populations or settings due to the specific context and characteristics of the study population.

## Figures and Tables

**Table 1:** Distribution of patients’ socio-demographic characteristics (*n* = 100).

Characteristics	*n*	(%)
**Gender**		
Male	20	(20%)
Female	80	(80%)
**Age**		
Less than 20	2	(2%)
21–40	38	(38%)
41–60	52	(52%)
61 and above	8	(8%)
**Nationality**		
Saudi	91	(91%)
Non-Saudi	9	(9%)
**Marital Status**		
Married	80	(80%)
Single	20	(20%)
**Occupation**		
Employed	35	(35%)
Not Employed	65	(65%)
**Education**		
Elementary	22	(22%)
Intermediate	6	(6%)
Secondary	21	(21%)
Bachelor’s	43	(43%)
Master’s	3	(3%)
Doctorate	5	(5%)

**Table 2:** Overall total median percent scores for quality of life of the studied patients.

Quality of Life Domains	Median	Interquartile Range
**Role functioning/emotional**	100	0
**Role functioning/physical**	100	25.0
**Pain**	100	0
**Social functioning**	100	0
**Emotional well-being**	92	12.0
**Physical functioning**	85	20.0
**Energy/fatigue**	80	25.0
**General health**	80	30.0

**Table 3:** Association between the socio-demographic or thyroid characteristics and the total mean quality of life scores for the studied patients.

Variables	Quality of Life (Mean ± SD)	Statistical Test (*p* Value)
**Gender**		
Male	87.0 ± 14.5	t = 1.022
Female	83.1 ± 15.3	*p* = 0.309
**Age**		
Less than 20	95.0 ± 7.1	F = 0.384 *p* = 0.765
21–40	84.1 ± 16.1	
41–60	83.2 ± 14.6	
61 and above	84.4 ± 16.5	
**Nationality**		
Saudi	83.5 ± 15.5	t = 0.918
Non-Saudi	88.3 ± 10.9	*p* = 0.361
**Marital Status**		
Married	84.6 ± 14.0	t = 0.872
Single	81.2 ± 19.3	*p* = 0.385
**Occupation**		
Employed	89.4 ± 9.4	t = 3.237
Not Employed	80.9 ± 16.8	*p* = 0.002 *
**Education**		
Elementary	71.8 ± 21.8	F = 5.852 *p* ≤ 0.001 *
Intermediate	76.7 ± 8.2	
Secondary	85.7 ± 12.2	
Bachelor’s	88.5 ± 9.2	
Master’s	100.0 ± 0	
Doctorate	89.0 ± 11.4	
**Indications for thyroidectomy**		
Benign thyroid diseases	83.4 ± 13.2	t = 0.615
Malignant thyroid diseases	84.4 ± 18.5	*p* = 0.543
**Type of thyroidectomy**		
Total Thyroidectomy	84.1 ± 15.3	t = 0.236
Thyroid lobectomy + Isthmusectomy	83.1 ± 15.1	*p* = 0.814

* *p* < 0.05—*Statistically significant*.

**Table 4:** Associations between the median percent general health scores of the studied patients and their socio-demographic or thyroid characteristics.

Variables	Median (IQR)	Statistical Test and *p* Value
**Gender**		
Male	82.5 (25.0)	Z = 0.654, *p* = 0.513
Female	80.0 (33.7)	
**Age**		
Less than 20	80 (10)	KW = 4.671, *p* = 0.198
21–40	82.5 (36.2)	
41–60	80.0 (30.0)	
61 and above	82.5 (21.2)	
**Nationality**		
Saudi	80.0 (30.0)	Z = 0.690, *p* = 0.490
Non-Saudi	75.0 (27.5)	
**Marital Status**		
Married	80.0 (28.7)	Z = 1.377, *p* = 0.168
Single	57.5 (57.5)	
**Occupation**		
Employed	85.0 (30.0)	Z = 1.925, *p* = 0.054
Not Employed	80.0 (30.0)	
**Education**		
Elementary	80 (37.5)	KW = 4.937, *p* = 0.424
Intermediate	85.0 (36.5)	
Secondary	75.0 (32.5)	
Bachelor’s	90.0 (30.0)	
Master’s	95.0 (10.0)	
Doctorate	85.0 (15.0)	
**Indication for thyroidectomy**		
Benign thyroid diseases	80.0 (35.0)	Z = 0.695, *p* = 0.487
Malignant thyroid diseases	82.5 (30.0)	
**Type of thyroidectomy**		
Total thyroidectomy	80.0 (30.0)	Z = 0.124, *p* = 0.902
Thyroid lobectomy + Isthmusectomy	80.0 (35.0)	

**KW**—Kruskal–Wallis test; **Z**—Mann–Whitney U test.

## Data Availability

Data are available on request from the authors.
